# Computational studies on non-nucleoside analogs of pyrimidine as NNRTIs against HIV-1

**DOI:** 10.1186/1471-2334-14-S3-E3

**Published:** 2014-05-27

**Authors:** Ritika Srivastava, Nidhi Singh, Anuradha Singh, Madhu Yadav, Ramendra K Singh

**Affiliations:** 1 Nucleic Acids and Antiviral Research Laboratory, Department of Chemistry, University of Allahabad, Allahabad (UP) - 211002, India

## Background

HIV is a retrovirus having ss RNA as the genetic material, which gets converted into ds DNA in the presence of reverse transcriptase (RT) enzyme. RT has been found the most attractive target for development of anti HIV-1 agents. Its inhibition is considered as one of the most practical approaches to prevent HIV infection.

## Methods

NNRTIs are non-competitive inhibitors and bind at allosteric site. Designing of non nucleoside analogs of pyrimidine as NNRTIs has been done on the basis of Lipinski’s Rule of Five & using the software DS 3.0. The molecules have been docked with HIV-RT using DS 3.0 in order to generate computational data and assess their suitability.

## Results

Docking experiments have shown good interaction of non nucleoside analogs of pyrimidine as NNRTIs. Analysis of the docking results revealed that these molecules adopted butterfly conformations while interacting at NNIBP of HIV-1 RT & formed hydrogen bonds with amino acids, Lys101, Lys103, Tyr181, Tyr318 and π-stacking interactions with Tyr181, Tyr188, Phe227 and Trp229.

## Conclusion

The molecular modeling revealed the interaction level of these molecules comparable to that of nevirapine. Hence, we predict these molecules as potential NNRTIs against HIV-1 RT.

